# Reliability and Spatial Consistency of MR Diffusion Tensor Imaging Measures Along the Cerebral Perivascular Space

**DOI:** 10.1111/jon.70086

**Published:** 2025-09-03

**Authors:** Enchao Qiu, Joga Chaganti, Phillip Phan, Mahdi Alizadeh, Devon Middleton, Kiran Talekar, Prabath K. Mondel, Scott H. Faro, Feroze B. Mohamed, Hsiangkuo Yuan

**Affiliations:** ^1^ Jefferson Headache Center, Department of Neurology Thomas Jefferson University Philadelphia Pennsylvania USA; ^2^ Jefferson Integrative Magnetic Resonance Imaging Center, Department of Radiology Thomas Jefferson University Philadelphia Pennsylvania USA; ^3^ Department of Neurosurgery Thomas Jefferson University Philadelphia Pennsylvania USA

**Keywords:** diffusion tensor imaging, diffusion tensor imaging analysis along the perivascular space, glymphatic system, observer variation, perivascular space

## Abstract

**Background and Purpose:**

Diffusion tensor imaging analysis along the perivascular space (DTI‐ALPS) has emerged as a promising noninvasive method for evaluating water motion that may reflect glymphatic system function. However, the reliability of DTI‐ALPS measurements across different region‐of‐interest (ROI) selection methods remains underinvestigated. This study aimed to assess the interrater reliability among three neuroradiologists in native space and compare DTI‐ALPS indices derived from ROIs placed in subjects’ native space versus standardized Montreal Neurological Institute (MNI) space.

**Methods:**

DTI‐ALPS indices from 16 healthy subjects were calculated from both left and right hemispheres using two ROI placement approaches: (1) native space ROIs manually placed by three neuroradiologists, and (2) standardized ROIs in MNI space based on the fractional anisotropy template. Interrater reliability was assessed using intraclass correlation coefficients (ICCs). The proportion of ROI overlaps among the three neuroradiologists was also evaluated. Differences between native and MNI space measurements were evaluated using related‐samples Friedman's analysis with post hoc pairwise comparisons.

**Results:**

Interrater reliability for native space ROI placement was moderate for left‐sided DTI‐ALPS indices (ICC = 0.599) and good for right‐sided DTI‐ALPS indices (ICC = 0.807). Spatial overlap analysis revealed poor Dice similarity coefficients across all ROI types (range: 0.047–0.312), with right association ROIs showing higher spatial consistency. Significant differences were found between native and MNI space measurements for left‐sided DTI‐ALPS indices (*p* = 0.002) but not for right‐sided DTI‐ALPS indices (*p* = 0.913).

**Conclusion:**

These findings highlight the importance of standardized ROI selection approaches for clinical applications of DTI‐ALPS.

## 1 Introduction

The glymphatic system, a perivascular fluid clearance pathway, plays a crucial role in eliminating waste products from the brain and has been implicated in various neurological disorders, including Alzheimer's disease [1], Parkinson's disease [2], and traumatic brain injury [3]. Diffusion tensor imaging analysis along the perivascular space (DTI‐ALPS) has emerged as a noninvasive method to evaluate glymphatic system function by measuring diffusion properties along perivascular spaces [4]. Since its introduction by Taoka et al. [4] in 2017, DTI‐ALPS has been increasingly utilized to investigate glymphatic dysfunction in various neurological conditions, including cerebral vascular diseases, headache disorders, epilepsy, and so forth [1, 5, 6, 7, 8, 9, 10, 11, 12, 13, 14, 15].

DTI‐ALPS calculations require the placement of regions of interest (ROIs) in projection and association fiber areas. These ROIs can be placed either in the subject's native anatomical space [4, 8, 16, 17, 18, 19] or in the standardized Montreal Neurological Institute (MNI) space [1, 6, 11, 20]. While native space ROIs may better reflect individual anatomical variations, MNI space ROIs offer standardization advantages for group comparisons and clinical convenience [21]. The reliability and spatial consistency of DTI‐ALPS measurements across different raters and ROI placement methods remain inadequately characterized. Previous studies have shown that ROI placement can be a significant source of variability in DTI metrics [22, 23], with potential implications for clinical interpretation and research reproducibility [24, 25]. While one recent study showed high consistency between manual native space and automated MNI space ROIs in 40 participants, this comparison was limited to automated methods and did not assess spatial overlap between manual placements by different raters [26].

This study aims to (1) assess the interrater reliability of DTI‐ALPS indices among three neuroradiologists using native space ROIs, (2) analyze the ROI differences in native space among the three neuroradiologists, and (3) compare DTI‐ALPS indices derived from ROIs placed in native space versus MNI space. By addressing these objectives, we seek to provide insights into the methodological considerations that may influence the standardization and clinical application of DTI‐ALPS measurements.

## 2 Methods

### 2.1 Study Participants

Sixteen healthy subjects (mean age: 28.8 ± 5.4 years; seven females) with no history of neurological or psychiatric disorders were recruited for this study. All participants provided written informed consent, and the study was approved by the institutional ethics committee in accordance with the Declaration of Helsinki.

### 2.2 Image Acquisition

Brain MRI images were acquired using a Siemens Prisma 3.0 Tesla MRI scanner equipped with a 64‐channel head–neck coil. The imaging protocol was as follows: *b*‐value = 1000 s/mm^2^ with 30 gradient directions, eight *b*_0_ images, slice thickness = 2.0 mm, voxel size = 1.8 × 1.8 × 2.0 mm^3^, slice acceleration factor = 2, 80 slices, repetition time/echo time = 5500/69 ms, field of view = 240 × 240 mm^2^, number of averages = 1, and acquisition time = 10 min. Additionally, TOPUP images with opposite phase encoding (posterior–anterior) direction were acquired to enable distortion correction.

### 2.3 DTI‐ALPS Processing

The DTI data were preprocessed using FSL (v 6.0, FMRIB Software Library, Oxford, UK, https://fsl.fmrib.ox.ac.uk/fsl) [27]. Preprocessing included eddy current correction, motion correction, and tensor fitting to generate fractional anisotropy (FA) and mean diffusivity maps. For each subject, two approaches were used for ROI placement: (1) Native Space approach: three neuroradiologists (J.C., P.K.M., and K.T.) with more than 10 years of experience in neuroradiology independently placed 5‐mm‐diameter spherical ROIs bilaterally in the projection area (predominantly in *x*‐axis direction) and association areas (predominantly in *z*‐axis direction) at the level of the lateral ventricle on each subject's color‐coded FA map, following the methodology described by Taoka et al. [4]. (2) MNI space approach: The subjects’ DTI data were transformed to the FMRIB58_FA template space using the FMRIB's Non‐linear Image Registration Tool (v 6.0.1, FMRIB Analysis Group, University of Oxford, Oxford, UK, http://fsl.fmrib.ox.ac.uk/fsl/fslwiki/FSL) [11]. Standardized MNI coordinate‐based selection was performed, utilizing spherical ROIs (5 mm diameter) placed on the registered FA template (FMRIB58_FA, 1 mm) at predetermined coordinates ([−27, −16, 29], [−39, −16, 29], [27, −16, 30], [39, −16, 30]) by a consensus of four neuroradiologists, two MR physicists, and two neurologists for left projection, left association, right projection, and right association fibers, respectively. Each ROI was then transferred to the individual's FA color map. Visual inspection confirmed proper ROI placement.

**FIGURE 1:**
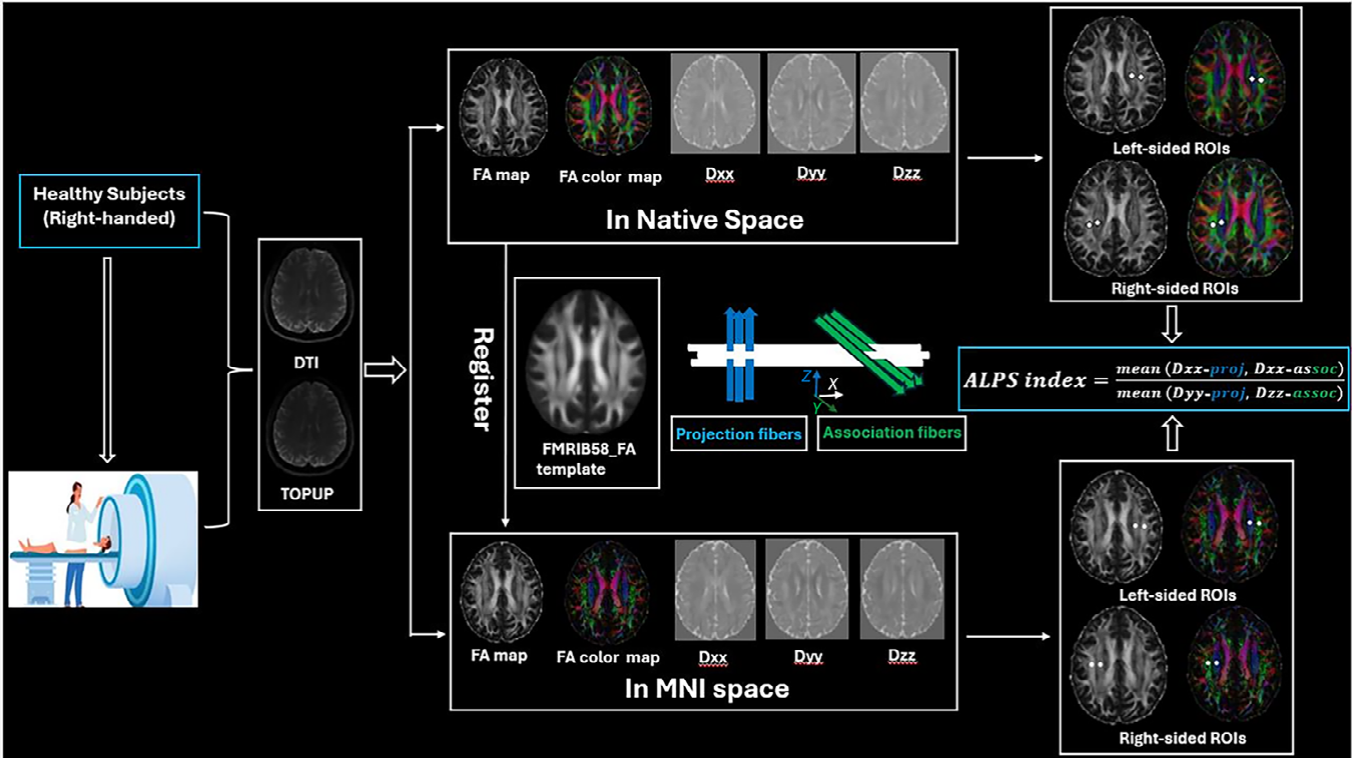
Calculation of diffusion tensor image analysis along the perivascular space (DTI‐ALPS) indices. This flowchart illustrates the DTI data acquisition and preprocessing, and processing for the bilateral DTI‐ALPS indices based on regions of interest (ROIs) in Native Space and Montreal Neurological Institute (MNI) space, respectively. DTI maps were transformed to MNI space from Native Space by registering to the functional magnetic resonance imaging of the brain (FMRIB) 58_FA template. TOPUP, diffusion‐weighted images acquired with posterior‐to‐anterior direction; FA, fractional anisotropy; Dxx: diffusivity along the *x*‐axis; Dyy: diffusivity along the *y*‐axis; Dzz: diffusivity along the *z*‐axis.

For both approaches, the DTI‐ALPS index was calculated according to the formula established by Taoka et al. [4], which represents the ratio between the diffusivity in projection and association fiber areas along specific axes:

$$\begin{array}{ccc} \mathrm{DTI}-\mathrm{ALPS}\mathrm{index} & = & \mathrm{mean}\left(\mathrm{Dxx}-\mathrm{proj},\mathrm{Dxx}-\mathrm{assoc}\right)/\\ & & \mathrm{mean}\left(\mathrm{Dyy}-\mathrm{proj},\mathrm{Dzz}-\mathrm{assoc}\right), \end{array}$$

where Dxx‐proj and Dyy‐proj are the *x*‐ and *y*‐components of diffusivity in the projection fiber area, and Dxx‐assoc and Dzz‐assoc are the *x*‐ and *z*‐components of diffusivity in the association fiber area. Figure 1 provides a comprehensive overview of the methodology employed in this study.

### 2.4 Statistical Analysis

Interrater reliability was assessed using the intraclass correlation coefficient (ICC) for measurements made by the three neuroradiologists in native space. The left‐versus‐right comparison for each rater was performed using the paired *t*‐test. The spatial consistency of ROI placement among raters was quantified using the Dice similarity coefficient, calculated as 2|*A*∩*B*|/(|*A*|+|*B*|), where *A* and *B* represent ROI masks from different raters. The comparison between native space and MNI space measurements was performed using related‐samples Friedman's analysis with post hoc pairwise comparisons. The coefficient of variation was calculated as the ratio of the standard deviation to the mean, expressed as a percentage, to assess measurement variability. Statistical significance was set at *p* < 0.05. All statistical analyses were performed using SPSS (v 27.0, IBM Corp., Armonk, NY, USA, https://www.ibm.com/spss).

## 3 Results

### 3.1 Interrater Reliability With ROI in Native Space

The interrater reliability of DTI‐ALPS measurements among the three neuroradiologists using native space ROIs is presented in Table 1. The ICC for left‐sided DTI‐ALPS indices was 0.599, indicating moderate reliability according to Cicchetti's criteria, while the ICC for right‐sided DTI‐ALPS indices was 0.807, suggesting good reliability. The coefficient of variation ranged from 6.45% to 9.96% across raters and hemispheres.

**TABLE 1:** The interrater reliability of left‐ and right‐sided diffusion tensor imaging analysis along the perivascular space indices in 16 healthy subjects with three neuroradiologists.

Native space	Rater 1	Rater 2	Rater 3	ICC
Left DTI‐ALPS index	1.570 ± 0.111 (7.07%)	1.494 ± 0.128 (8.57%)	1.504 ± 0.097 (6.45%)	0.599
Right DTI‐ALPS index	1.577 ± 0.146 (9.26%)	1.557 ± 0.155 (9.96%)	1.586 ± 0.156 (9.84%)	0.807

*Note*: Data are presented as median (interquartile range) or mean ± standard deviation (coefficient of variation).

Abbreviations: ICC, intraclass correlation coefficient; DTI‐ALPS, diffusion tensor imaging analysis along the perivascular space.

### 3.2 Comparison of Left‐ and Right‐Sided DTI‐ALPS Indices for Each Rater

Table 2 presents the comparison of left‐ versus right‐sided DTI‐ALPS indices for each rater using paired *t*‐tests. No statistically significant differences were observed between the left and right sides across all raters. The correlation coefficients ranged from –0.160 to 0.432, indicating low to moderate, nonsignificant associations between hemispheric measurements.

**TABLE 2:** Comparison of the left‐sided diffusion tensor imaging analysis along the perivascular space indices versus the right‐sided for each rater using paired *t*‐test.

Left vs. right	Correlation	*p* value
Rater 1	−0.160	0.554
Rater 2	0.432	0.094
Rater 3	0.213	0.427

### 3.3 Spatial Overlap Analysis of ROI Placement

**FIGURE 2:**
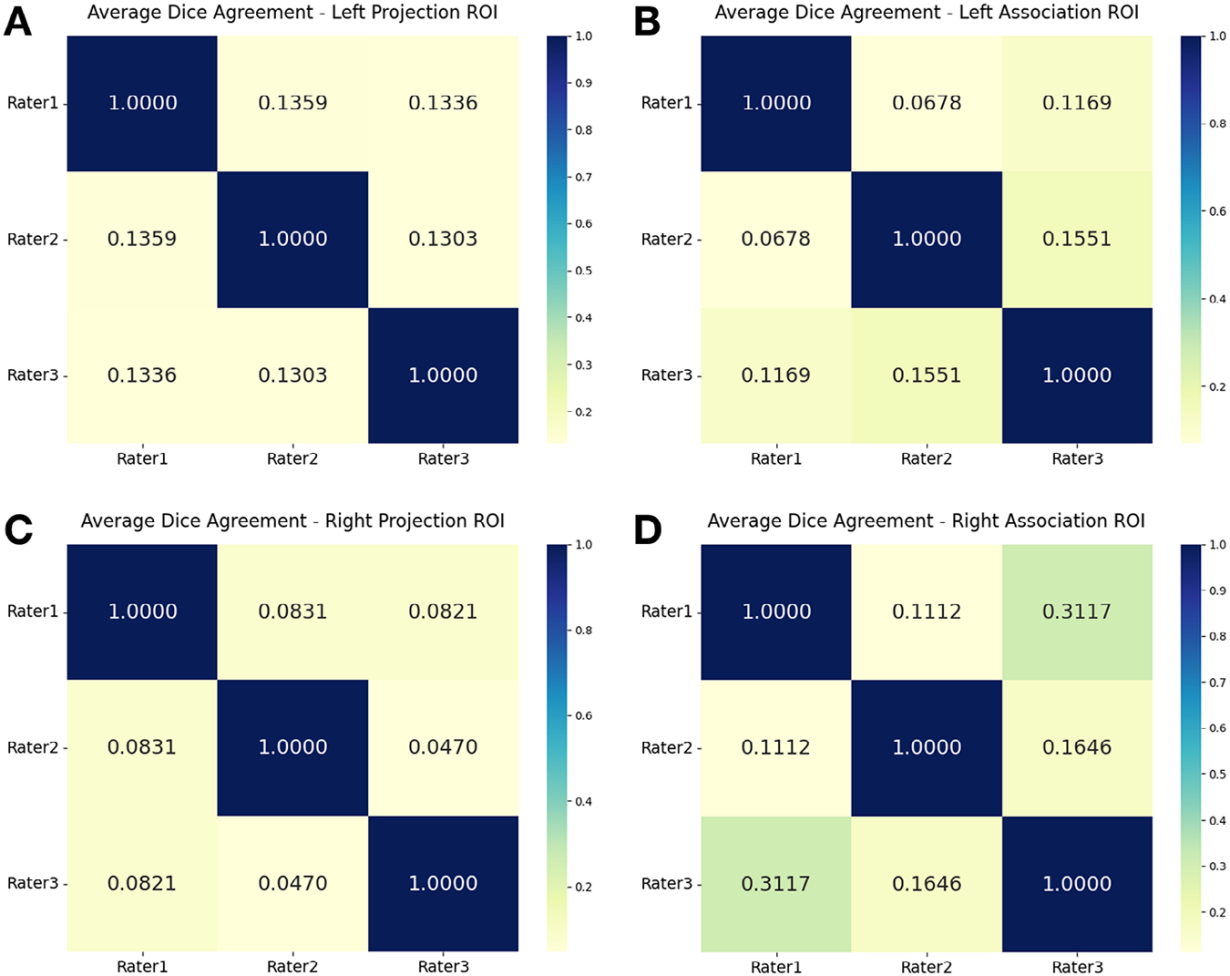
Spatial overlap analysis of diffusion tensor image analysis along the perivascular space region of interest (ROI) placement across three raters. Heatmaps showing Dice similarity coefficients for pairwise comparisons among three neuroradiologists (Rater 1, Rater 2, Rater 3) for four ROI categories: (A) left projection ROIs, (B) left association ROIs, (C) right projection ROIs, and (D) right association ROIs. Color scale represents Dice coefficient values from 0 (yellow, no overlap) to 1.0 (dark blue, perfect overlap). Diagonal elements represent perfect self‐agreement (1.0000). Values indicate the spatial similarity of ROI placement between rater pairs, with higher values representing better spatial consistency.

The spatial consistency of ROI placement across the three raters was evaluated using Dice similarity coefficients for each ROI type (Figure 2). Overall, the Dice coefficients demonstrated poor spatial overlap across all ROI categories, with values consistently below 0.35 for all rater pairs.

### 3.4 Comparison of Native Space and MNI Space Measurements

The DTI‐ALPS indices were compared across ROIs in native space with different raters and across ROIs in MNI space using a related‐samples Friedman's analysis. For left‐sided DTI‐ALPS indices, a statistically significant difference was observed (*p* = 0.002), with pairwise comparisons revealing significant discrepancies between the native space with Rater 2 and the MNI space (*p* = 0.010), and between the native space with Rater 3 and the MNI space (*p* = 0.004). However, no significant differences were found among the three raters for ROI in native space (e.g., Rater 1 vs. Rater 2, *p* = 0.792), suggesting consistency among native‐space raters. In contrast, right‐sided DTI‐ALPS indices showed no significant differences across all comparisons (*p* ≥ 0.913), demonstrating high reproducibility irrespective of rater or spatial reference. These findings highlight the potential variability introduced by spatial normalization when analyzing left‐sided DTI‐ALPS metrics. Table 3 shows the details.

**TABLE 3:** Comparison of diffusion tensor imaging analysis along the perivascular space indices from regions of interest placed in native space (Rater 1, 2, 3) versus Montreal Neurological Institute space.

		Friedman pairwise comparisons					
DTI‐ALPS indices	Related‐samples Friedman's analysis	Rater 1 and Rater 2	Rater 1 and Rater 3	Rater 2 and Rater 3	Rater 1 and MNI space	Rater 2 and MNI space	Rater 3 and MNI space
Left sided	*p* = 0.002	*p* = 0.792	*p* = 0.450	*p* = 1.000	*p* = 0.602	*p* = 0.010	*p* = 0.004
Right sided	*p* = 0.913	*p* = 1.000	*p* = 1.000	*p* = 1.000	*p* = 1.000	*p* = 1.000	*p* = 1.000

Abbreviations: DTI‐ALPS, diffusion tensor imaging analysis along the perivascular space; MNI, Montreal Neurological Institute.

## 4 Discussion

This study evaluated the interrater reliability of DTI‐ALPS indices when measured in native space, quantified the spatial concordance among three radiologists through Dice coefficient analysis across four different ROI locations, and compared DTI‐ALPS indices obtained from native space versus MNI space frameworks. Our findings reveal important considerations for standardizing DTI‐ALPS methodology and highlight the variability inherent in manual ROI placement across different raters and coordinate systems.

### 4.1 Interrater Reliability of DTI‐ALPS Measurements

The interrater reliability analysis revealed moderate to good agreement among neuroradiologists for DTI‐ALPS measurements, with notable hemispheric asymmetries in measurement consistency. Specifically, right‐sided ALPS measurements showed superior interrater reliability compared to left‐sided measurements, suggesting that anatomical landmarks or measurement protocols may be applied with greater consistency in the right hemisphere. This hemispheric asymmetry in measurement reliability merits further investigation, as it may reflect either inherent neuroanatomical differences in perivascular space organization or systematic biases in ROI placement techniques [28].

The observed coefficients of variation indicate reasonable but not exceptional measurement precision across raters. These values are comparable to other DTI‐based biomarkers reported in the literature [24], though they highlight the need for standardized training protocols and potentially automated or semiautomated ROI placement methods to improve consistency [29].

### 4.2 Spatial Overlap Analysis of ROI Placement

The Dice coefficient analysis provides crucial insights into the spatial consistency of ROI placement across raters, revealing systematic patterns that complement the DTI‐ALPS reliability findings. The observed poor spatial overlap among raters indicates substantial variability in ROI placement despite efforts to standardize anatomical landmarks. Notably, the right association ROIs showed relatively better spatial agreement compared to other regions; this aligns with the superior reliability values observed for right‐sided ALPS measurements. The particularly low Dice coefficients for projection fiber ROIs suggest that these anatomical regions may be more challenging to identify consistently, possibly due to their smaller size or less distinct boundaries on DTI images.

The asymmetry between left and right hemisphere ROI agreement mirrors the reliability patterns observed in the ALPS measurements themselves. Left projection and association ROIs showed similar low‐level agreement, while right‐sided ROIs demonstrated more variable agreement patterns, with association ROIs achieving notably higher overlap than projection ROIs. Nevertheless, no significant differences were observed between left and right DTI‐ALPS indices for any rater. This discrepancy may reflect region‐specific asymmetry effects, where differences in anatomical or microstructural organization at certain locations influence ROI reproducibility without altering the overall ALPS index values [30].

### 4.3 Impact of MNI Space on DTI‐ALPS Measurements

The comparison between native space and MNI space measurements revealed significant differences specifically for left‐sided ALPS indices, while right‐sided measurements remained consistent across coordinate systems. Importantly, MNI space ROI selection eliminates interrater variability entirely, as standardized coordinates are used consistently across all measurements, representing a major methodological advantage for cross‐study comparisons and meta‐analyses. This standardization contrasts sharply with native space approaches, where our results demonstrated poor spatial overlap among experienced raters, reflecting the inherent subjectivity in manual ROI placement that MNI space methods effectively eliminate.

The hemispheric differences observed between native and MNI space measurements likely reflect both subjective selection variations among raters and underlying neurobiological asymmetries. The superior reliability of right‐sided measurements (ICC = 0.807) compared to left‐sided measurements (ICC = 0.599) in native space suggests that raters may find left‐hemisphere anatomical landmarks more challenging to identify consistently, possibly due to inherent structural complexity or subtle anatomical variations. This interpretation is supported by neurobiological evidence demonstrating interhemispheric white matter asymmetries, including leftward asymmetry in the arcuate fasciculus and other association tracts, as well as asymmetries in projection fibers such as the corticospinal tract [31, 32]. Additionally, Schilling et al. [33] revealed that “radial asymmetry is widespread throughout white matter” and that “crossing fibers significantly inflate ALPS indices,” suggesting that the left hemisphere may exhibit greater microstructural complexity. This complexity may render manual ROI placement more variable among raters while making these regions more sensitive to the geometric transformations in spatial normalization procedures [34].

### 4.4 Clinical and Methodological Implications

The observed variations in DTI‐ALPS measurements, combined with the poor spatial overlap in ROI placement, have several important implications for clinical research and application that extend beyond simple measurement error to fundamental questions about methodological standardization, accuracy, and cross‐study comparability. The elimination of interrater variability through MNI space ROI selection represents a critical advancement for establishing DTI‐ALPS as a reliable biomarker, as the poor spatial consistency demonstrated in native space approaches would severely compromise the reproducibility required for clinical translation and multicenter studies. Although the MNI approach demonstrates superior precision and standardization, the fundamental question of accuracy—whether MNI or native space measurements better reflect true glymphatic function—remains unresolved and requires validation through direct pathological correlation studies.

The hemispheric differences in measurement reliability reveal that anatomical complexity and rater subjectivity interact in ways that differentially affect left and right hemisphere assessments, with potential implications for lateralized pathological processes. The relatively superior reliability of right‐sided measurements correlates with better spatial agreement observed for right association ROIs, suggesting that certain anatomical regions may be more reliably identifiable across raters, while the moderate reliability of left‐sided measurements may reflect genuine challenges in consistently identifying complex anatomical landmarks rather than random measurement error. This asymmetric reliability pattern has important consequences for studies investigating hemispheric differences in glymphatic function, as differential measurement precision between hemispheres could introduce systematic bias in the detection of lateralized pathological changes, regardless of which spatial framework ultimately proves more accurate. The clinical relevance of such hemispheric differences is demonstrated by recent studies showing asymmetric glymphatic dysfunction in temporal lobe epilepsy and preferential left‐hemisphere impairment in early Parkinson's disease [35, 36].

The standardization advantages of MNI space approaches become particularly critical when considering the clinical application of DTI‐ALPS indices across diverse populations and scanning protocols, yet these benefits must be weighed against the unknown trade‐offs in biological accuracy. While native space approaches may theoretically better capture individual anatomical variations and preserve subject‐specific glymphatic architecture, the substantial interrater variability observed suggests that this theoretical advantage is negated by practical measurement inconsistencies that would undermine clinical utility. Conversely, the consistent application of standardized MNI coordinates facilitates the development of normative databases and clinical cutoff values essential for clinical translation, but future validation studies incorporating direct measures of glymphatic function, cerebrospinal fluid flow dynamics, or postmortem pathological assessment will be necessary to determine whether the precision gains achieved through MNI standardization translate to improved accuracy in detecting true glymphatic dysfunction.

### 4.5 Limitations and Future Directions

Several limitations should be acknowledged in interpreting these results. The sample size was adequate for reliability assessment but may not explore the age‐ or gender‐related differences or capture the full range of variability expected in clinical populations. Additionally, the study focused on healthy individuals, and reliability may differ in pathological conditions where perivascular space architecture is altered.

The low Dice coefficients observed across all ROI types raise important questions about the current state of DTI‐ALPS methodology. While the reliability values suggest acceptable performance for group‐level analyses, the poor spatial overlap indicates that individual measurements may be highly dependent on rater‐specific ROI placement strategies. This disconnect between functional reliability and spatial consistency suggests that current DTI‐ALPS measurements may be capturing signal from anatomically variable regions while still providing clinically relevant information.

Furthermore, validation studies in clinical populations with known perivascular space pathology would help establish whether the observed measurement variability affects the clinical utility of DTI‐ALPS indices. The relationship between spatial overlap and measurement reliability should be further explored to determine acceptable thresholds for both metrics in clinical applications.

## 5 Conclusion

This study demonstrates that DTI‐ALPS ROI selections are associated with interrater reliability with important hemispheric differences. Our findings suggest that while native space approaches may better capture individual anatomical variations, the substantial interrater variability undermines their practical utility, whereas MNI space methods eliminate rater‐dependent variability entirely, providing superior standardization essential for clinical applications. However, the fundamental question of whether standardized MNI or native space ROIs better reflect true glymphatic function remains unresolved and requires future validation through direct pathological correlation studies.

## Conflicts of Interest

M.A. received funding from NIH (R01NS134987). F.M. received funding from NIH (R01NS111113). Within the past 24 months, H.Y. received funding from the American Headache Society Early‐Stage Investigator Research Award; institutional support for serving as an investigator from Teva, AbbVie, Ipsen, Parema, Shiratronics, and Pfizer; consultant fees from Salvia, AbbVie, Pfizer, and Cerenovus; and royalties from Cambridge University Press and MedLink. The other authors declare no conflicts of interest.
